# Presence of immune cells, low tumor proliferation and wild type *BRAF* mutation status is associated with a favourable clinical outcome in stage III cutaneous melanoma

**DOI:** 10.1186/s12885-017-3577-x

**Published:** 2017-08-29

**Authors:** Johan Falkenius, Hemming Johansson, Rainer Tuominen, Marianne Frostvik Stolt, Johan Hansson, Suzanne Egyhazi Brage

**Affiliations:** Department of Oncology-Pathology, Karolinska Institutet, Cancer Center Karolinska, Karolinska University Hospital, 171 76 Solna, Stockholm Sweden

**Keywords:** Ki67, Immune cells, *BRAF* mutation, Prognosis, Stage III melanoma

## Abstract

**Background:**

The variable prognosis in stage III cutaneous melanoma is partially due to unknown prognostic factors. Improved prognostic tools are required to define patients with an increased risk of developing metastatic disease who might benefit from adjuvant therapies. The aim was to examine if cellular immune markers in association with tumor proliferation rate and *BRAF* mutation status have an impact on prognosis in stage III melanoma.

**Methods:**

We have used two sets of case series with stage III disease: 23 patients with short survival (≤ 13 months) and 19 patients with long survival (≥ 60 months). Lymph node metastases were analyzed for Ki67, CD8 and FOXP3 protein expression using immunohistochemistry. *BRAF* mutation status was analyzed in a previous study on the same samples.

**Results:**

Low tumor proliferation rate was significantly associated with a better prognosis (*p* = 0.013). Presence of FOXP3+ T cells was not correlated to adverse clinical outcome. A highly significant trend for a longer survival was found in the presence of an increasing number of markers; CD8+ and FOXP3+ T cells, low tumor proliferation and *BRAF* wildtype status (*p* = 0.003). Presence of at least three of these four markers was found to be an independent favorable prognostic factor (OR 19.4, 95% CI 1.9-197, *p* = 0.012), when adjusting for ulceration and number of lymph node metastases. Proliferation alone remained significant in multivariate analyses (OR 26.1, 95% CI 2.0-344, *p* = 0.013) but with a wider confidence interval. This panel still remained independent when also adjusting for a previously identified prognostic glycolytic-pigment panel.

**Conclusions:**

We have demonstrated that presence of immune cells in association with tumor proliferation and *BRAF* mutation status may further contribute to identify stage III melanoma patients with high risk of relapse.

## Background

The known favorable prognostic factors in stage III cutaneous melanoma (CMM) according to the AJCC classification, including micrometastasis, low number of lymph node metastases, and no ulceration of the primary tumor, are insufficient to accurately predict clinical outcome [1, 2]. There is thus a need for additional prognostic markers to more reliably determine the risk for progressing from regionally advanced CMM (stage III) to disseminated CMM (stage IV). Although recent breakthroughs in therapy have led to more options to treat patients with metastatic CMM and a higher probability for long-term survival for a subset of patients, patients with disseminated stage IV disease are still not regarded as curable. It is therefore of great importance to prevent progression from regionally advanced CMM in stage III to further disseminated stage IV disease.

Several gene expression studies have reported a correlation between overexpressed immune related genes in stage III-IV CMMs and a good clinical outcome [3–5] including a previous study by us [6]. In our microarray-based study a number of gene ontology (GO) categories related to immune response showed a significantly higher expression among long-term compared to short-term survivors. A similar finding was also observed in a proteomic study on stage III CMM [7].

In recent years there has been a lot of interest in immunological prognostic markers due to the paradigm shift with immunotherapy which has led to improved progression-free and overall survival among CMM patients with disseminated disease [8–12]. For example, tumor-associated antigens NY -ESO-1, PD-L1 and PD-L2 have been correlated with clinical outcome in metastatic CMM, including stage III – IV disease [13–15] but no molecular markers are yet validated for clinical use to predict risk for development of stage IV disease.

In general, a strong immunological host response is considered to be prognostically favorable. Lardone et al. compared three independent gene expression studies and found a common immune gene signature of T cell-associated genes among favorable outcome patients with stage III-IV CMM [16]. Several studies have demonstrated that presence of tumor infiltrating lymphocytes (TILs) in primary tumors is associated with a better clinical outcome [17, 18]. It has been reported that a high number of CD4+ and CD8+ TIL cells in metastatic CMM lesions is associated with better clinical outcome, particularly in tumors with high CD8+ content [19]. However, a discordant report, suggested that TILs in primary thin CMMs with regression phenomena may promote progression and metastasis [20]. Thus, the prognostic value of TILs needs to be further investigated and is not included in the current AJCC staging system [1]. So far there are few studies on TIL’s prognostic role in stage III CMM, but data supports a favorable association [21].

Forkhead box P3 (FOXP3) is an important transcriptional regulator of the differentiation and immunosuppressive function of regulatory T-cells (Tregs) and has been used as a marker for Treg activity. FOXP3+ Tregs are in general associated with an unfavorable clinical outcome [22] but in recent years publications have shown that they can sometimes be a favorable marker for clinical outcome [23]. The prognostic role of FOXP3+ Tregs thereby becomes more complex.

Ki67 is widely utilized as a proliferation marker for many cancers, but is still not used routinely in CMM. However, there are several studies correlating an elevated Ki67-index in primary tumors with a poor prognosis in CMM, suggesting that Ki67-index may be a useful prognostic marker also in CMM [24–26]. Transciptome studies on stage III-IV CMM have shown that a proliferative gene signature is associated with a worse clinical outcome [3, 4].

The mitogen-activated protein kinase (MAPK) pathway plays a pivotal role in regulation of cell proliferation by involving a series of protein kinase cascades, including the ERK cascade, with BRAF as one key kinase [27]. Approximately 50% of CMM tumors harbor an activating *BRAF* mutation in codon 600 (>90% of these are *BRAF V600E*) [28]. Some studies have demonstrated that a *BRAFV600E* mutation has adverse prognostic impact; while others have not been able to confirm this [29–32]. So far the prognostic value of carrying an activating *BRAF* mutation is thus unclear.

Recently immunotherapy with the checkpoint inhibitor ipilimumab received approval for adjuvant therapy in stage III CMM by the US Federal Drug Agency [33]. More adjuvant therapies are expected in near future, hence addressing the increased need for prognostic markers to identify patients suitable for adjuvant therapy in stage III CMM.

Although potential immunological and proliferative prognostic markers have been identified from transcriptome and proteomic studies there is still a gap regarding the prognostic impact of these biomarkers. The aim of the present study was to examine the impact of immune cells in association with the Ki67 proliferation marker and *BRAF* mutation status on clinical outcome in stage III CMM using two series of patients with distinctly separated clinical outcome, in order to identify a novel panel of prognostic markers.

## Methods

### Patients and tumor specimens

We have used two sets of case series described in our previous study consisting of totally 42 patients with macrometastatic stage IIIB-C disease, according to the current 7th edition of AJCC cancer staging, selected on the basis of time from regional lymph node dissection to death or last follow-up [6]. We studied 23 patients with short survival, ≤ 13 months and 19 patients with long survival ≥60 months. The patients underwent lymph node dissection between 1994 and 2002 at the Karolinska University Hospital and specimens were collected in a biobank. A biopsy from each lymph node dissection was formalin fixed and paraffin embedded.

The cause of death was generalized CMM for all cases in the short-term survival group. Three of the long-term survivors showed relapses with locoregional recurrence within five years and one of them started palliative chemotherapy five years after lymph node surgery.

Eight of the short-term survivors received adjuvant radiotherapy (*n* = 6) or interferon therapy (*n* = 2). Among the long-term survivors three subjects received adjuvant radiotherapy (*n* = 1) or interferon therapy (*n* = 2). Data was missing for three cases.

### Immunohistochemistry

Immunohistochemistry was performed on 4-μm-thick, formalin-fixed, paraffin-embedded sections. Heat-induced antigen retrieval was performed for all the antibodies in a decloaking chamber according to the manufacturer’s instructions (Biocare, Concord, CA, USA). We used EDTA buffer pH 9 for CD8 and FOXP3 and citrate buffer pH 6 for Ki67. Endogenous peroxidase was blocked with 3% hydrogen peroxide for 10 min at room temperature. Sections were washed 3X in TBS buffer, blocked for unspecific binding by incubation in 2.5% horse serum for 20 min at room temperature and incubated overnight at 4 °C with the primary monoclonal mouse antibodies. Anti-human CD8 1:100 (clone C8/144B) (Dako, Denmark), FOXP3 1:100 (clone 236A/E7) (eBioscience,AffymetrixCompany, USA) and Ki67 1:200 (clone MIB-1) (Dako, Denmark) were used. Negative controls were incubated without the primary antibody. Secondary antibody incubation using streptavidin/peroxidase complex was done according to a kit manual (Vectastain Universal Quick Kit, Vector Laboratories Inc., CA, USA), as was development with DAB Substrate Kit(Vector Laboratories Inc) for developing and counterstained with Mayers Hematoxylin (Histolab,Sweden).

Independent evaluation of all slides was performed by three observers (S.E.B, J.F, M.F-S). In case of discrepancies between observers, a consensus was reached on further review. CD8 and FOXP3 expression were evaluated by assessing percentage of cells with nuclear and cytoplasmic staining in five separate 1hpf/hotspot areas and calculating the mean value. Ki67 was evaluated by estimating percentage of tumor cells with nuclear staining in the whole section. The ranges of values were 0-100% for FOXP3, 5-90% for Ki67 and 0-30% for CD8. The median percentage of CD8 and FOXP3 positive cells for all cases was used as a cut-off between low and high expression. The proportion of Ki67 positive tumor cells was estimated and a cut-off level of 25% was used.

### *BRAF* mutation analysis

DNA extraction and mutation screening was performed by pyrosequencing (*n* = 29) using PSQ.

HS 96A Pyrosequencer (Biotage, Uppsala, Sweden) or by direct Sanger sequencing (*n* = 13) using an ABI Prism 310 automated sequencer (Applied Biosystems, Foster City, California, USA) as reported previously [6, 30]. Both technologies are able to detect all varieties of V600 mutations.

### Statistical analysis

Differences in bivariate associations between clinicopathological variables, mutational status and staining parameters were tested using Fisher’s exact test. For continuous data *p*-values were calculated using the Mann-Whitney test.

Logistic regression was used for test of trend. Logistic regression was also used to evaluate the effect of different biomarkers on clinical outcome. Results are presented with odds-ratio (OR) and 95% confidence interval (95% CI). All *p*-values are two-sided and refer to Wald tests. All analyses were carried out using the statistical software STATA version 14.1 (Stata Corp LP, College Station, Texas, USA). No correction for multiple testing has been performed.

## Results

### Characteristics of the two case series

Overall 42 patients were included in the study: 23 with short survival (≤ 13 months) and 19 with long survival (> 60 months). Details about patients and pathological characteristics are shown in Table 1. Breslow thickness, ulceration and the number of lymph node metastases were significantly different between the two groups, with more unfavorable characteristics such as thicker and more ulcerated primary tumors and more lymph nodes involved in the short survival group. There were no significant differences between the two prognostic groups regarding gender and *BRAF* mutation status but more patients in the short survival group had tumors carrying *BRAF* mutation than the long survival group, 56% and 32%, respectively (Table 1) [6]. In total 45% (19/42) of the cases were carrying a *BRAF* mutation. Among patients with tumors carrying a *BRAF* mutation 95% (18/19) had a *BRAF* V600E and 5% (1/19) had a *BRAF* V600 K.

**Table 1:** Baseline patient and pathological characteristics modified from reference 6

Characteristic	Short (%)	Long (%)	*P*-value^a^
Gender			
Male	14 (61)	13 (68)	
Female	9 (39)	6 (32)	0.75
Breslow thickness (mm)			
Mean + SD	4.9 + 6.1	1.9 + 1.6	0.016
Ulceration			
Present	15 (65)	3 (16)	
Absent	8 (35)	16 (84)	0.002
Number of metastatic nodes			
1	5 (22)	13 (68)	
2-3	14 (61)	3 (16)	
> 4	4 (17)	3 (16)	0.005
Mutation status:			
BRAF mutated			0.13
V600E	12(52)	6 (32)	
V600 K	1 (4)	−	
BRAF wildtype	10 (43)	13 (68)	

^a^ *P*-values calculated using Fisher’s exact test for categorical data and Mann-Whitney test for continuous data

### CD8 and FOXP3

**Fig. 1:**
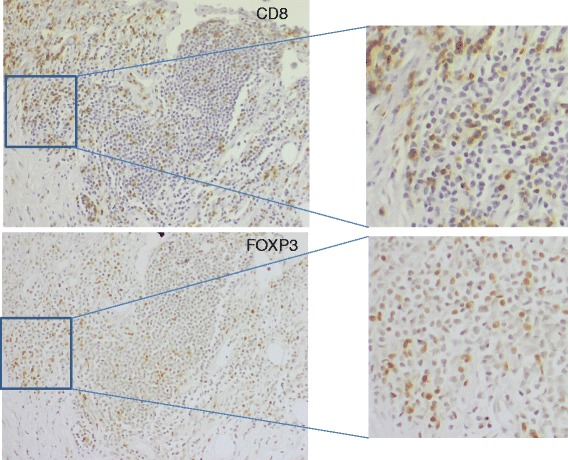
Immunohistochemistry performed on serial sections of a CMM regional lymph node metastasis. Expression of CD8+ (a) and FOXP3+ (b) T cells is shown

Representative images of CD8+ and FOXP3+ staining are shown in Fig. 1. More tumors among long-term survivors had high CD8+ expression compared to tumors among short-term survivors, 63% (12/19) and 35% (8 /23), respectively and this difference was borderline significant (*p* = 0.07). A statistically non-significant difference in the proportion of FOXP3+ cells was observed in tumors from long survivors compared to tumors from short survivors: 63% (12/19) and 43% (10/23), respectively (*p* = 0.21).

There was a significant positive correlation between CD8 and FOXP3 protein expression in T cells (*p* = 0.029). No significant correlation to favorable clinical outcome was observed when we created a three-level variable of no markers, one of two markers or both markers positive, respectively (*p* = 0.09).

When comparing all CD8+ and FOXP3+ hotspot areas there seems to be an overlap suggesting that some T-cells may be both FOXP3+ and CD8+ as shown in Fig. 1. Thirteen samples showed positive expression for both CD8 and FOXP3 and in 11 of the cases there was a varying degree of overlap observed. In five samples a major overlap >50% was observed. In one of the samples no overlap was observed between the hotspots and in one other sample the analysis was not performed on adjacent sections.

We did not find any association between *BRAF* mutation status [6] and expression of CD8+ T-cells in our material (*p* = 0.23).

### Proliferation

**Fig. 2:**
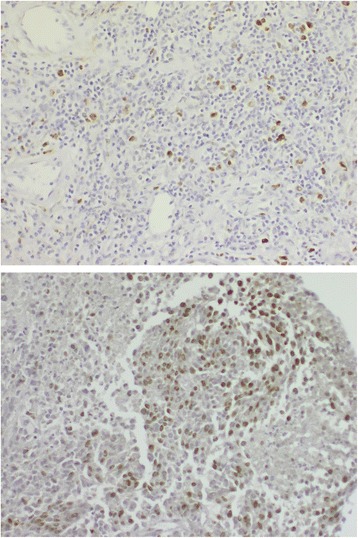
Immunohistochemistry performed on two regional CMM lymph node metastases. Expression of protein Ki67 is demonstrated. The upper panel shows a tumor with low proportion (below 25%) of Ki67 staining, while the lower panel shows one with a high proportion (above 25%)

We investigated tumor proliferation by Ki67 staining in the two case series and found a significantly higher proportion of Ki67 positive tumors among short survivors (*p* = 0.013) as shown in Table 2. Twenty one percent (4/19) of the long survivors and 61% (14 /23) of the short survivors had tumors with high proportions of Ki67 staining cells. The median % positive cells were 17.5% among long survivors (range 5-90%) versus 27.5% among short survivors (range 5-50%). Representative images of tumor samples with low or high Ki67 staining are illustrated in Fig. 2. None of the long survivors had a tumor exhibiting both a *BRAF* mutation and high Ki67 positivity, compared to 30% (7/23) of the short survivors (*p* = 0.011).

**Table 2:** Survival groups by favourable biomarker status

Factor	Short survival	Long survival	Unadjusted		Adjusted^c^	
			OR^a^ (95% CI)	*P* ^b^	OR^a^ (95% CI)	*P*
BRAF						
Wildtype	10 (43)	13 (57)	2.8 (0.8-10)	0.11	4.5 (0.7-27)	0.10
Mutation	13 (68)	6 (32)	1		1	
Ki67						
Low	9 (38)	15 (63)	5.8 (1.5-23)	0.013	26.1(2.0-344)	0.013
High	14 (78)	4 (22)	1		1	
FOXP3						
Low	13 (65)	7 (35)	1	0.21	1	0.42
High	10 (45)	12 (55)	2.2 (0.6-7.7)		1.9 (0.4-9.4)	
CD8						
Low	15 (68)	7 (32)	1	0.071	1	0.17
High	8 (40)	12 (60)	3.2 (0.9-11)		3.0 (0.6-15)	
Number of favourable markers out of the above 4						
At least 3	4 (25)	12 (75)	8.1 (2.0-34)	0.004	19.4 (1.9-197)	0.012
Less than 3	19 (73)	7 (27)	1		1	

^a^Odds-ratio and 95% confidence interval estimated using unconditional logistic regression

^b^Wald test *p*-value

^c^Controlling for ulceration and LGLL

### Combining CD8, FOXP3, Ki67 and *BRAF*

**Fig. 3:**
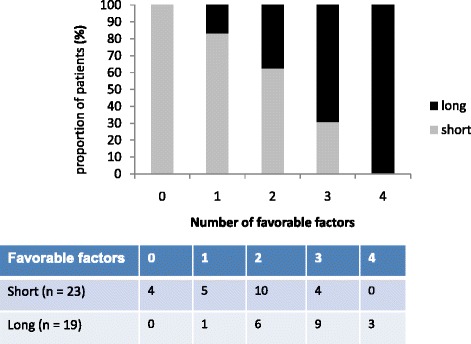
The prognostic power of a panel consisting of potentially four favorable prognostic markers, including high expression of CD8+ T-cells and FOXP3+ T-cells, low tumor expression of Ki67 and *BRAF* wildtype status is demonstrated. A highly significant increase in the proportion of long-term survivors in association with the increase from one to four favorable markers (Logistic regression, *p* = 0.003)

We wanted to investigate if a panel of potentially favorable prognostic markers, including high expression of CD8+ T-cells and FOXP3+ T-cells, low expression of Ki67 and *BRAF* wildtype status has a higher prognostic impact compared to Ki67 alone. We performed a test of trend and found a highly significant increase in the proportion of long-term survivors by including several potentially favorable markers in the analysis (*p* = 0.003), as presented in Fig. 3, supporting the relevance of using a panel of multiple prognostic markers. The best result in univariate analysis was found when at least three of four markers were present, which gave an odds ratio of 8.1 (95% CI 2.0-34) for belonging to the group with long survival (*p* = 0.004).

### Multivariate analysis

Presence of ulceration in the primary tumor and number of lymph node metastases are included in the current 7th edition of AJCC melanoma staging system as parameters for classifying patients in sub-stages IIIA-C. The best result in our panel of markers, with at least three of four factors present, remained significant in the multivariate analysis, when adjusting for ulceration and number of macroscopic lymph node metastases (OR 19.4, 95% CI 1.9-197, *p* = 0.012) as shown in Table 2. Ki67 alone remained significant in the multivariate analysis (OR 26.1, 95% CI 2.0-344, *p* = 0.013) but had a wider 95% CI in comparison to the combined index. The 8th edition of AJCC melanoma staging will be implemented from January 2018 and will also include Breslow thickness. We have therefore also considered Breslow in the multivariate analysis. Our panel of markers still remained significant (OR 17.7, 95% CI 1.67-188, *p* = 0.017) after adjusting for Breslow thickness, ulceration and number of macroscopic lymph node metastases.

In our previous study based on gene expression profiling but validated at the protein level, on the same sets of case series, we presented a prognostic panel consisting of three glycolytic proteins (GAPDHS, GAPDH, PKM2) and one pigment synthesis-related protein (TYRP1) where a positive expression of at least two out of four proteins was found to be an independent adverse prognostic factor for clinical outcome (*p* = 0.011) [6]. When also including this panel in the multivariate analysis and adjusting for ulceration and number of nodal macroscopic metastases the results remained significant for both the current (OR 64.0, 95% CI 2.5-1672, *p* = 0.012) and previous (OR 48.3, 95% CI 2.6-888, *p* = 0.009) panels indicating that they are independent of each other.

## Discussion

In this study we have demonstrated that a panel consisting of candidate prognostic markers related to immune response (expression of FOXP3+ and CD8+ T-cells), proliferation (Ki67 expression) and *BRAF* mutation status, can give significant prognostic information. Ki67 was the only single marker showing a significant difference between the patients with long versus short clinical outcome (*p* = 0.013) but presence of at least three of four favorable markers was a stronger prognosticator of favorable clinical outcome in the univariate analysis (*p* = 0.004) and remained significant in the multivariate analysis (*p* = 0.012). In accordance with our previous gene expression profiling study a panel of several biomarkers thus demonstrates a stronger correlation to clinical outcome in stage III melanoma than any single marker. In both studies there were two series of patients with distinctly separated clinical outcome and excluding an intermediate survival group, which could be considered as a limitation.

In a previous publication [6] we demonstrated that a panel of markers was better than any marker alone in prognostication of clinical outcome in stage III CMM, indicating the importance to focus on activity in multiple gene pathways and biological functions rather than single genes or proteins. When adding our previously identified prognostic panel (glycolytic-pigment) to the multivariate analysis, both panels remained significant. Hence, the impact of the two different prognostic panels is independent of each other and supports the importance of focusing on multiple gene pathways and biological functions rather than single genes or proteins. One contributing explanation for this is the commonly observed intra and inter tumor heterogeneity in CMM [34].

There is a limited overlap between gene expression studies on stage III-IV CMM regarding specific genes, possibly related to differences in study design, selection of patients from different CMM stages, as well as in array platforms [3–5]. These studies have however identified similar GO categories of interest for prognostic impact, for example related to immune response and proliferation. A strategy to overcome the problem with tumor heterogeneity could be to use a combination of different prognostic variables, including clinicopathological variables, to better predict clinical outcome [35].

A gene expression study has also been performed in stage IIIA CMM analyzing a cohort of positive sentinel lymph nodes and demonstrated that genes related to immune response were differentially expressed between patients with a good and poor prognosis [36]. This supports the value of assessing the expression of immune response proteins also in stage IIIA CMM to identify patients with a higher risk of relapse already at time of diagnosis.

Ki67 is a proliferative marker which is associated with adverse clinical outcome in several malignancies, but not used routinely in CMM. There are several publications on Ki67 in stage I-II CMM demonstrating a prognostic adverse impact [24–26]. So far there is limited published data on Ki67 in stage III CMM. Our result gives support for including Ki67 in a panel of prognostic markers for stage III CMM. In addition, high Ki67 expression appears by itself to have a stronger impact than low numbers of CD8+ T cells or presence of *BRAF* mutation in our study, which may be important to take into consideration when prognosis is assessed.

The prognostic impact of harboring an activated *BRAF* mutation in CMM is still unclear, with discordant results in different studies, and thus needs to be further investigated [29–32]. Our results show that *BRAF* mutation has an adverse prognostic impact in combination with other unfavorable factors, but not by itself (Table 1). This observation is supported by findings from other studies showing an adverse prognostic impact of *BRAF* mutation when co-existing with other prognostic markers. There is a study by Mann et al. demonstrating that *BRAF* mutation, *NRAS* mutation and absence of immune-related expressed genes are associated with adverse prognosis in stage III CMM [37]. Recently co-occurrence of *TERT* promoter and *BRAF* mutation has been associated with a more aggressive clinical behavior in CMM [38]. Altogether, these data indicate that the prognostic impact of *BRAF* mutations is dependent on additional factors.

It has recently been demonstrated that inhibition of mutated *BRAF* leads to induction of CD8+ T cell infiltration [39] suggesting that *BRAF* mutated tumors may directly or indirectly suppress CD8+ T cells. However, we did not find any correlation between *BRAF* mutation status [6] and expression of CD8+ T cells in our material.

Today there are no established predictive markers for immunotherapy with antibodies against CTLA-4 and PD-1 in CMM. However, there are several studies indicating that both preexistence and a post-treatment increase in intratumoral CD8+ T cells in metastatic CMM may predict response to therapy with CTLA-4 and PD-1 blockade [40–42]. We found a significant positive correlation between CD8+ and FOXP3+ T-cells, in concordance with another recent study on a cohort including stage III CMM patients with sentinel lymph node metastases [43].

FOXP3 is an important regulator of differentiation and immunosuppressive function of Tregs. However, FOXP3 expression is not restricted to Tregs as different tumor cells, including CMM cells, express the protein, and also to some extent CD8+ T-cells [44]. In general, FOXP3+ Tregs are associated with an unfavorable clinical outcome but we found that a high expression of FOXP3+ T cells was more common among long survivors than short survivors, although not statistically significant. Furthermore, Churlaud et al. found an increase of FOXP3+ CD8+ T regulatory cells (Tregs) following interleukin-2 stimulation [45], implicating that there could be a predictive relevance of Treg cells with FOXP3 and CD8 co-expression. In this study we have documented that there seems to be an overlap of FOXP3+ and CD8+ suggesting that some T-cells may be both FOXP3+ and CD8 + .

In other studies on colorectal, gastric and breast cancer there has also been a positive correlation between high expression of FOXP3 and favorable clinical outcome [23]. The discordant results regarding the prognostic value of FOXP3 may be due to the presence of four isoforms of human FOXP3, with different suppressive roles on T effector cells [46, 47]. The FOXP3 antibody used in this study recognizes both the full length protein and the exon 2 splicing variant lacking suppressive ability and it is therefore uncertain whether the FOXP3+ T cells in our investigation are suppressive or lack this ability. It is assumed that FOXP3 may generally have a suppressive role but appears as a favorable prognostic marker in some cancers because of its association with CD8+ T cells [23]. Our results support this assumption.

## Conclusions

Although our two patient sets were small, which is a limitation, the results demonstrate the prognostic relevance of CD8+ and FOXP3+ T cells, tumor proliferation rate and *BRAF* mutation status, and support the concept that several factors in combination predict prognosis in stage III CMM better than single markers. The design of this study with two extreme groups regarding prognosis could be considered as an advantage to identify potential prognostic markers. However, further validation on an independent unselected cohort including an intermediary prognostic group is needed to confirm these findings. Today there are several treatment options against metastatic melanoma disease, and recently a checkpoint inhibitor was approved by FDA for adjuvant therapies, supporting the clinical need for a prognostic panel to identify patients in stage III CMM with a high risk of relapse, who may benefit from adjuvant therapy.

## Abbreviations

95% CI: 95% confidence interval
CMM: Cutaneous melanoma
FOXP3: Forkhead box P3
GO: Gene ontology
MAPK: Mitogen-activated protein kinase
OR: Odds-ratio
TILs: Tumor infiltrating lymphocytes
Tregs: Regulatory T-cells
